# Using a Mobile Phone App to Identify and Assess Remaining Stocks of *In Situ* Asbestos in Australian Residential Settings

**DOI:** 10.3390/ijerph16244922

**Published:** 2019-12-05

**Authors:** Matthew Govorko, Lin Fritschi, Alison Reid

**Affiliations:** School of Public Health, Curtin University, Perth 6845, Western Australia; matthew.govorko@curtin.edu.au (M.G.); Lin.Fritschi@curtin.edu.au (L.F.)

**Keywords:** asbestos, identification, mobile phone application, residential environment

## Abstract

Asbestos-containing materials (ACMs) were used extensively throughout much of the 20th century and can still be found in many Australian homes. Therefore, we developed a mobile application (“app”), called ACM Check, which guides users through a home inspection to identify and assess certain types of *in situ* ACM. A cross-sectional study was conducted using the app to collect data on the type and condition of *in situ* asbestos in Australian residential settings. Since being released in June 2017, we have received data for 702 home inspections. Of these, 578 (82.3%) houses contained a total of 1895 *in situ* materials categorised as positive for asbestos by the app. The most prevalent ACMs were used for the backing board to electrical meter boxes (50% of homes), eaves and soffit linings (44.2% of homes), and fencing (28.1% of homes). While the majority of ACMs were categorised as ‘very low’ or ‘low’ priority for removal or remediation, 6.6% of all ACMs were considered to be of ‘high’ priority. Mobile apps offer a platform to help increase people’s awareness of possible health hazards found in the residential environment, such as asbestos, while also being used to collect data for public and environmental health research.

## 1. Introduction

Asbestos is a commercial term encompassing a family of naturally occurring fibrous silicate minerals with a crystalline structure. This includes the three most commercially significant asbestos fibre types, chrysotile, crocidolite and amosite, as well as actinolite, anthophyllite and tremolite. Asbestosis, benign pleural disease, lung cancer and malignant mesothelioma are diseases related to asbestos exposure [1,2,3].

Globally, Australia is the country that consumed the highest amount of asbestos on a per capita basis during the 1950s and 1960s [4,5]. Australia mined asbestos as early as the 1880s and manufactured asbestos cement products from the 1920s until the late 1980s [6,7]. The asbestos cement manufacturing industry accounted for 60% of all production (i.e., mining of raw asbestos) and 90% of all consumption (i.e., consumption equals production plus imports minus exports) of asbestos fibres in Australia [7,8]. By 1954, Australia was the fourth highest gross consumer of asbestos cement products globally, only behind the United States, United Kingdom, and France [7]. Asbestos-containing materials (ACMs) were commonly used in Australian residential settings for such things as roofing, eaves, exterior and interior wall cladding, and fencing [9]. Up until the 1960s, 25% of new Australian homes were clad with asbestos-cement products [7]. It is currently estimated that one third of all Australian homes contain asbestos products and it is highly likely all homes built before the mid-1980s contain at least one ACM [6]. 

In Australia, asbestos-cement materials were phased out of use in the 1980s, and a total ban on asbestos use of any type, including a prohibition on the importation, manufacturing, processing, sale, storage and re-use of asbestos and ACMs was implemented from 31 December 2003. Despite the prohibition, a large reservoir of asbestos remains *in situ* as a lasting legacy of Australia’s past use. Individuals continue to be exposed to asbestos when these *in situ* ACMs are disturbed, such as through maintenance, renovations, and demolition of the building, or when the condition of the ACM deteriorates [10,11,12,13,14]. However, *in situ* ACMs are difficult to identify. Previous surveys have indicated that although the majority of Australian do-it-yourself home renovators and members of the general public believed that ACMs are common in Australian buildings, there was a considerable proportion who had no confidence in their ability to identify asbestos and did not know what types of materials contain asbestos [15,16,17]. 

A primary recommendation arising from the *National Asbestos Profile for Australia* was that “there is an identified need for more research to gain a better understanding of the amount and location of ACMs in the residential sector” [18] (p. 39). However we do not know the amount of ACM remaining *in situ* in the residential environment or the current condition of these ACMs. Such knowledge is needed to make appropriate decisions concerning either its prioritised removal or its containment. 

For those reasons we developed [19], tested [20], and released a specifically designed mobile phone application (“app”), ACM Check, which guides users through a visual inspection of the interior and exterior of the home in order to identify certain types of ACM remaining *in situ*. This app has two main purposes: (1) to increase the user’s awareness and knowledge of potential ACMs present in the residential environment so that appropriate safety precautions can be used when dealing with the material; and (2) to collect data from consenting users regarding the type and condition of *in situ* ACM still found in Australian residential settings. The aim of this paper is to demonstrate how a mobile app can be used to estimate the prevalence and condition of ACMs remaining in the Australian residential environment.

## 2. Materials and Methods 

### 2.1. Study Design

A cross-sectional study was conducted between June 2017 and September 2019 that involved individuals taking the following steps: (1) downloading the mobile app, ACM Check, onto their mobile device from the App Store (Apple Inc., Cupertino, CA, USA) or Google Play (Google Inc, Mountain View, CA, USA), (2) consenting to share their questionnaire data and participate in the study, and (3) completing the app’s questionnaire on an Australian residence. Data were only collected via ACM Check from consenting users. Individuals who downloaded the app and opted not to share their data still had full use of the app, but no information was collected from them. The study was approved by Curtin University’s Human Research Ethics Committee (RDHS-89–15).

### 2.2. Participant Recruitment

Curtin University distributed a media release on 13 June 2017, which began the recruitment campaign. The media release was circulated to a range of organisations in Australia related to asbestos, cancer prevention and awareness, occupational health and safety, and public and environmental health. Radio interviews, articles in local newspapers, posts on social media platforms (i.e., Twitter and Facebook), and advertisements on an online classifieds and community website were used to promote the app and the study. Additionally, approximately 500 recruitment flyers were delivered to homes located in the Perth metropolitan area. Non-Western Australian participants were recruited through items posted on social media, webpages or distributed via subscription newsletters or emails. 

The inclusion criteria for the study were that individuals were aged 18 years or older, had access to either an Android device or an iOS device (e.g., iPhone, iPod Touch or iPad) with iOS version 8 or newer installed, and completed the app on an Australian residence. In order to participate, users had to click the toggle button to the “on” position on the first screen of ACM Check, which indicated that “I have read the information statement and consent to participate in the research.”

### 2.3. Data Collection

All data collection occurred through the mobile app, ACM Check, between 13 June 2017 and 14 September 2019. A detailed description of the design and development of ACM Check has been published elsewhere [19]. In short, the app contains a questionnaire that guides users through a visual inspection of 14 key locations inside and outside of a residential building. The areas inspected for ACM include the exterior walls, eaves/soffit lining, roofing, gutters, downpipes, electrical meter box, fencing, outbuilding walls, outbuilding roofs, interior walls and splash backs, backing to wall tiles, ceilings, flooring, and heater flues. Questions ask about the age of the dwelling, renovation history, and key characteristics of the building materials used. ACM Check then uses the answers to automatically classify each material/category into one of four probabilities of containing asbestos (‘not applicable,’ ‘unlikely,’ ‘possible,’ or ‘likely’ ACM). Moreover, the term ‘possible’ ACM is used to classify the circumstances where it is more difficult to confirm or rule out the probability that a material contains asbestos. This can be due to such difficulties as an absence of visual characteristics that distinguish ACM from non-ACM or a lack of information on the year of installation or renovation history.

For each ‘possible’ or ‘likely’ ACM, the user is asked to rate the current condition (‘very poor,’ ‘poor,’ ‘fair,’ or ‘good’) and its potential for disturbance (‘unlikely,’ ‘somewhat likely,’ ‘likely,’ or ‘highly likely’). Each response option is accompanied by descriptive text to assist the user in rating the material (see Table S1 for list of ratings and descriptive text). The two ratings are automatically assigned numerical values and added together by ACM Check to give an overall priority level to each ACM (‘very low,’ ‘low,’ ‘moderate,’ or ‘high’ priority). The priority level indicates the ACMs that are of most significance regarding the potential risk of asbestos exposure at each property, and, in turn, informs the user on a general course of action to minimise the risk (see Table S1).

At the completion of the inspection, the app automatically saves all questionnaire results to a secure external server in comma separated format (.csv). Furthermore, each user can complete the inspection on one or more houses.

### 2.4. Validity of ACM Check

Before the app was released to the public and implemented as a data collection tool, a cross-sectional study was conducted testing the app on a sample of 40 homes built before 1990 located in the metropolitan region of Perth, Western Australia (WA). The results are published elsewhere [20]. The participants completed the app on their home and the results were compared to the findings of onsite inspections conducted by an environmental consultant. Agreement between the two methods, determined using Cohen’s kappa values, ranged from fair to substantial when categorising the different areas as positive or negative for asbestos. In particular, the tool overestimated the occurrence of asbestos in the category ‘wall tile backing.’ The app was therefore altered so that the highest ACM rating that could be assigned to wall tile backing was ‘possible.’ The category was kept in the app to make users aware of the possibility of asbestos exposure when removing old wall tiles due to asbestos previously being used in mastics and adhesives. However for the current study, all positive ratings for wall tile backing were classified as negative.

### 2.5. Statistical Analysis

All statistical analyses were completed in IBM SPSS Statistics for Windows, Version 26 (IBM Corp., Armonk, NY, USA). There were originally four rating outcomes for the probability a material contained asbestos, either ‘not applicable,’ ‘unlikely,’ ‘possible,’ or ‘likely’ ACM. ‘Possible’ and ‘likely’ responses were coded as ‘positive’ for asbestos while ‘not applicable’ and ‘unlikely’ responses were coded as ‘negative’ for asbestos (see Table S2 for the frequencies of ‘possible’ and ‘likely’ ACM ratings before they were combined as ‘positive’). 

In regard to the age of the dwelling, the user must select one of three age categories including ‘Before 1985,’ ‘Between 1985 and 1990,’ and ‘After 1990.’ If the user selects ‘After 1990,’ then the user completes an abbreviated questionnaire that only includes an assessment of the fencing and outbuilding roofs and walls (as these may be present from previous developments). All other materials are automatically labelled as ‘not applicable’ or ‘unlikely’ by the app and thus labelled as ‘negative’ for the analysis.

The houses were separated by location into WA and non-WA in order to examine if there were any differences in the prevalence and condition of *in situ* ACMs found in WA compared with other regions of Australia. Chi-square and Mann-Whitney U tests were undertaken to identify differences by location (WA vs. non-WA houses). 

## 3. Results

### 3.1. User and Housing Characteristics

ACM Check was downloaded 1466 times between June 2017 and September 2019. The user agreed to share data for a total of 702 inspections, of which 336 (47.9%) were for inspections completed in WA whilst the rest were completed in other Australian states and territories (n = 366; 52.1%). Most non-WA participants were from Victoria (n = 189; 26.9%), New South Wales (n = 75; 10.7%), and Queensland (n = 59; 8.4%), the most populous Australian states. ‘Community members’ made up two-thirds (67%) of users in WA and over half (56.6%) of users in other Australian states and territories (Table 1)

The majority of houses screened in WA were separate houses (86.6%), had 1–2 (39.6%) or 3–4 (38.5%) occupants, and two-thirds were built pre-1985 (66.4%). These characteristics were similar for inspections occurring in other Australian states and territories (Table 1). However, a higher percentage of houses screened in WA were built after 1990 compared with houses screened in other states and territories (17.3% vs. 9.8%, respectively; Table 1). 

### 3.2. Prevalence of Asbestos-Containing Materials

Of the 702 houses inspected by participants using ACM Check, 578 (82.3%) had at least one type of material that was categorised as positive for asbestos. A total of 549 (78.2%) homes had at least one material categorised as positive for asbestos located outside and 333 (47.4%) had at least one positive material located inside (Table 2). A total of 1,895 materials were categorised by ACM Check as positive for asbestos with the majority located outside the house (n = 1311; 69.2%). Moreover, the majority of these positive ACMs were initially categorised as ‘likely’ ACM (n = 1559; 82.3%) by ACM Check (see Table S2).

Of the 336 WA houses, 276 (82.1%) had at least one suspected ACM located anywhere on the property with 60 (17.9%) houses being free of ACM. Two hundred and seventy (80.4%) houses had an ACM located outside and over one-third (n = 132; 39.3%) had an ACM located inside (Table 2). Across the 276 WA houses with suspected ACM, there was a total of 855 materials categorised as positive for asbestos with three-quarters of these located outside (n = 646; 75.5%).

In WA, the most common ACMs were flat asbestos-cement sheeting used as the backing board to the electrical meter box and corrugated asbestos-cement sheet fencing, which were categorised as positive by ACM Check at over half of the properties (54.5% and 50.6%, respectively; Table 2). In addition, 75 (44.1%) of the 170 users with asbestos–cement fences indicated that some or all of the sections of fencing also had asbestos-cement capping. With regards to the amount of corrugated asbestos-cement sheet fencing present on the property, 77 (45.3%) users reported that they had fewer than 25 asbestos-cement sheets, while 50 (29.4%) users had 26-50, 28 (16.5%) users had 51–75, 10 (5.9%) users had 76-100, and 5 (2.9%) users had 100 or more asbestos-cement sheets in place. 

Furthermore, 131 (39%) WA houses had flat asbestos-cement sheeting used for the eaves, soffit lining, verandah ceilings and/or carport ceilings. The least common ACMs found in this sample of WA houses were asbestos-cement gutters (n = 6; 1.8%) and corrugated asbestos-cement sheeting used for outbuilding roofs (n = 21; 6.3%; Table 2). 

For houses in other Australian states and territories, the most common forms of ACMs were flat asbestos-cement sheeting used for the eaves, soffit lining, verandah ceilings and/or carport ceilings (n = 179; 48.9%), and flat asbestos-cement sheeting used for the backing board to electrical meter boxes (n = 168; 45.9%). A considerably lower percentage of houses in other states and territories had asbestos-cement sheet fences compared with houses in WA (7.4% vs. 50.6%, respectively). Similar to WA, the least common category of ACM in other Australian houses was asbestos-cement gutters (n = 18; 4.9%; Table 2 [21]).

### 3.3. Priority Assessment of Suspected ACMs

#### 3.3.1. Current Condition and Potential for Disturbance Ratings

Of the 855 ACMs in WA houses, the majority were rated as being in either ‘good’ (n = 353; 41.3%) or ‘fair’ (n = 357; 41.8%) condition. Over one in ten ACMs (n = 121; 14.2%) were rated as being in ‘poor’ condition by WA users of ACM Check. The majority of ACMs in ‘poor’ condition were located outside (n = 98/121; 81%) with fencing (n = 39/121; 32.2%) and eaves (n = 17/121; 14%) being the most frequently rated ACMs in ‘poor’ condition. Overall, only 24 of the 855 ACMs (2.8%) in WA houses were rated as being in ‘very poor’ condition. However, nearly a quarter of identified ACMs in other Australian States and Territories were assessed as being in ‘poor’ (n = 167/1040; 16.1%) or ‘very poor’ (n = 79/1040; 7.6%) condition (see Table S3). The current condition ratings were significantly poorer for electrical meter box backing boards and interior walls in houses located in other states and territories than in WA. Conversely, current condition ratings for heater flue pipes were worse in WA than in other states.

The majority of suspected ACMs in WA houses were rated as ‘unlikely’ (n = 416/855; 48.7%) or ‘somewhat likely’ (n = 283/855; 33.1%) to be disturbed in the near future. Nearly ten percent of suspected ACMs were rated as ‘likely’ to be disturbed (n = 81/855; 9.5%), with fencing being the most commonly reported ACM ‘likely’ to be disturbed (n = 24) across the WA houses. A larger proportion of ACMs in other states were rated by users as ‘likely’ (14.8%) or ‘highly likely’ (13.6%) to be disturbed compared with ACMs in WA houses (9.5% and 8.8%, respectively; Table S3). The potential for disturbance ratings were significantly higher for ACM roofing in other states and territories than in WA. Of the 45 ACM roofs in other states and territories, 15.6% (n = 7) were rated ‘highly likely’ and 22.2% (n = 10) were rated ’likely’ to be disturbed, whereas only one of the 23 ACM roofs in WA were rated as ‘highly likely’ and none were rated as ‘likely’ to be disturbed. The potential for disturbance ratings for all other ACM categories did not show differences by location.

#### 3.3.2. Priority Levels for WA Houses

Of the 336 WA houses, 28 (8.3%) had one or more materials categorised as ‘high’ priority for removal or remediation and 106 (31.5%) houses had at least one ACM categorised as ‘moderate’ priority. When looking at only the positive materials (n = 855) in WA houses, approximately 20% (n = 168; 19.6%) were categorised as ‘moderate’ priority while only 3.9% (n = 33) of ACMs were categorised as ‘high’ priority by ACM Check. Fencing and interior flooring had the highest number of ‘high’ priority ACMs (Table 3).

#### 3.3.3. Priority Levels for Other Australian Houses

For the 366 houses screened in other Australian states and territories, 46 (12.6%) contained one or more ‘high’ priority ACMs and 123 (33.6%) had at least one ACM present that was categorised as ‘moderate’ priority. Regarding only the positive materials (n = 1040) in other Australian houses, close to one-quarter of positive materials were categorised as ‘moderate’ priority (n = 239; 23%) while 8.8% (n = 92) were categorised as ‘high’ priority (Table 4). Asbestos cement guttering, interior flooring, and asbestos cement sheet roofing (either on the house or outbuilding) had the highest proportion of ‘high’ priority ACMs (Table 4).

The priority levels for gutters, drainpipes, electrical meter box backing board and interior walls in other states were significantly higher than in WA. For example, regarding interior walls, 12.4% were rated as ‘high’ priority and 27.8% ‘moderate’ priority in other Australian houses compared with none and 25%, respectively, in WA houses. The priority levels for all other ACM categories did not show differences by location.

## 4. Discussion

Since ACM Check was launched in June 2017, 702 people have used the app to systematically screen the inside and outside of a house for the presence of *in situ* ACM and consented to share their data. Of these, 578 (82.3%) houses contained a total of 1,895 *in situ* materials that were categorised as positive for asbestos by the app. It is evident from the results that ACMs are still prevalent in the Australian residential environment. The majority of *in situ* asbestos was located outside the home with flat asbestos-cement sheeting used as the backing board to electrical meter box, flat asbestos-cement sheeting used for eaves, and corrugated asbestos-cement sheet fencing being the most frequently detected ACMs. 

One of the major differences between the type of ACMs in WA houses compared with houses in other Australian states and territories was the occurrence of corrugated asbestos-cement sheet fencing, which was much more prevalent around the houses assessed in WA than around houses in other states and territories (50.6% vs. 7.4%, respectively). In contrast, a higher percentage of the identified ACMs were located inside the home in other Australian states and territories than in WA (36.1% vs. 24.4% of all ACMs were located inside, respectively). The lower occurrence of ACMs inside WA houses may be due to the fact that more houses built post-1990 were inspected in WA than in other Australian states and territories (17.9% vs. 9.8%, respectively; Table 1). The manufacture of asbestos-cement products and their use in residential buildings was phased out during the 1980s and houses built since then are unlikely to contain ACMs [18]. However, ACMs can still be found on properties where the house was built after 1990 as older asbestos-cement fences or outbuilding structures could still be present from previous developments. 

Of note, potential for disturbance ratings were significantly higher for ACM roofing in other Australian states and territories than in WA. If ACM roofing is unlikely to be removed in WA, then the material will continue to be present in the residential environment with an ongoing potential to release fibres. Whereas in other states and territories, the higher potential for disturbance ratings may be related to the planned removal of the ACM roofing in the near future, which then comes with the associated risks of asbestos exposure during the removal process. 

The results indicate that the majority of ACMs identified in the WA and other Australian state and territory houses screened were of ‘very low’ (n = 920/1,895; 48.5%) or ‘low’ (n = 443/1,895; 23.4%) priority for remediation or removal. However, there were 125 (6.6%) ‘high’ priority ACMs with the most frequent being interior flooring and asbestos-cement sheeting used for eaves, soffit linings and/or verandah ceilings. Furthermore, there was a higher percentage of houses containing at least one ‘high’ priority ACM in other Australian states and territories compared with WA (12.6% vs. 8.3% of houses, respectively). The difference could possibly be explained by the higher proportion of inspections completed by users who were environmental health officers (EHOs) in other Australian states and territories compared to in WA (15.6% vs. 11%, respectively). EHOs may be attending or screening more ‘at-risk’ properties than the average community member due to the nature of their job. In addition, our pilot study showed that ACM Check users tended towards overestimating the priority of ACMs in and around their home (i.e., they frequently rated materials as being in poorer condition or having a higher potential for disturbance than did the environmental consultant) [19]. As such, it is unlikely that these results are underestimating the priority levels of the ACMs screened in this sample. 

A limitation of the study is the sample size. A larger sample size would have provided greater information regarding the prevalence of *in situ* ACMs and their condition in the residential environment. Nonetheless, we believe that the sample still provides an indication of the most common ACMs remaining in Australian residential settings. For instance, the sample suggests that corrugated asbestos-cement sheet fencing, flat asbestos-cement sheet eaves, and the backing board to old electrical meter boxes are still prevalent in the Western Australian and wider Australian housing stock. A benefit of using this type of mobile app as a research tool is that data collection can be ongoing. Therefore, a larger sample size can be obtained, provided the app is maintained and sufficiently promoted. 

A second limitation of the study is that the sample population was self-selected, and this can lead to selection bias. Moreover, there is a lack of data on age, sex and socioeconomic status of participants who complete the app, which means that we could not standardize the data to provide truly representative data for the country. Individuals who already know where ACMs are located in their home may be unlikely to take the time to download and complete the app, resulting in either a lower response rate or an underestimation of the prevalence of ACM in our analysis. Conversely, these same individuals may be more likely to download and complete the app because they know that asbestos is present and want to contribute to the study. Thirdly, it is possible that the priority levels in this study are overestimated considering that the users of ACM Check tended to overrate ACMs in comparison to the environmental consultant’s ratings in the pilot study [22]. Finally, an assumption when analysing the data is that all submitted results are from genuine inspections and not simply the user testing the app.

In Australia, community members’ (including general public, do-it-yourself home renovators, and tradespeople) self-rated confidence in their ability to identify ACM has remained relatively stable over the last three years the national asbestos awareness survey has been conducted. This suggests that the task of communicating the range of materials that could contain asbestos in residential settings continues to be an important and ongoing one [17]. Moreover, younger do-it-yourself home renovators (18–39 years), females, those currently renting, and those from non-English speaking backgrounds indicated lower ratings regarding feeling informed about asbestos and its dangers and their confidence in being able to identify ACMs compared with other groups [17]. Therefore, there is still a clear need for asbestos awareness campaigns in Australia that target these particular segments of the community as well as the general public. Future campaigns should look to promote the uptake of digital resources, such as ACM Check, together with promoting the message using conventional media (e.g., websites, television, social media platforms) to raise asbestos awareness in Australia and elsewhere. 

## 5. Conclusions

Despite new ACMs being phased out of use in residential buildings during the 1980s, there remains a large reservoir of *in situ* asbestos in the Australian residential environment. However, the amount and condition of the ACMs remaining in the Australian housing stock is unknown. Our study shows that a specially designed mobile phone app, ACM Check, can be used by a range of community members to collect data on the presence, current condition, and potential for disturbance of *in situ* asbestos in Australian residential settings. This is the first such mobile phone app and questionnaire to be trialed in this population. Based on data collected using ACM Check, the most prevalent *in situ* ACMs were used for the backing board to electrical meter boxes, eaves and soffit linings, and fencing. While the majority of ACMs were categorised as ‘very low’ or ‘low’ priority for removal or remediation, ten percent of all houses in the sample contained at least one ‘high’ priority ACM. Mobile apps offer a platform to help increase people’s awareness of possible health hazards found in the residential environment, such as asbestos, while also being used to collect data for public and environmental health research.

## Figures and Tables

**Table 1:** Demographic information for users who completed ACM Check.

Factor	Western Australia	Other	Total
**User description**			
Community member	225 (67%)	207 (56.6%)	432 (61.5%)
Environmental health officer	37 (11%)	57 (15.6%)	94 (13.4%)
Licensed asbestos removalist	14 (4.2%)	12 (3.3%)	26 (3.7%)
Tradesperson, labourer, handyperson	60 (17.9%)	90 (24.6%)	150 (21.4%)
**Period built**			
Before 1985	223 (66.4%)	270 (73.8%)	493 (70.2%)
Between 1985 and 1990	55 (16.4%)	60 (16.4%)	115 (16.4%)
After 1990	58 (17.3%)	36 (9.8%)	94 (13.4%)
**Type of dwelling**			
Separate house	291 (86.6%)	308 (84.2%)	599 (85.3%)
Flat, unit or apartment	22 (6.5%)	32 (8.7%)	54 (7.7%)
Semi-detached, row, villa, terrace house or townhouse	23 (6.8%)	26 (7.1%)	49 (7%)
**Number of occupants**			
0 ^1^	25 (6.8%)	27 (8%)	52 (7.4%)
1–2	145 (39.6%)	134 (39.9%)	279 (39.7%)
3–4	141 (38.5%)	130 (38.7%)	271 (38.6%)
5+	55 (15%)	45 (13.4%)	100 (14.2%)
**Total users**	336 (100%)	366 (100%)	702 (100%)

^1^ This includes houses that have no current occupants such as houses that are on the market or set for demolition.

**Table 2:** Western Australian and other Australian state and territory houses with materials categorised as ‘positive’ for asbestos by ACM Check.

Category	Western Australia (n = 336)	Other (n = 366)	Total (n = 702)
**Outside**			
Exterior wall cladding	54 (16.1%)	72 (19.7%)	126 (17.9%)
Eaves	131 (39%)	179 (48.9%)	310 (44.2%)
Roof	23 (6.8%)	45 (12.3%)	68 (9.7%)
Gutters	6 (1.8%)	18 (4.9%)	24 (3.4%)
Downpipes	31 (9.2%)	57 (15.6%)	88 (12.5%)
Backing board to electrical meter box	183 (54.5%)	168 (45.9%)	351 (50%)
Fencing	170 (50.6%)	27 (7.4%)	197 (28.1%)
Outbuilding walls	27 (8%)	55 (15%)	82 (11.7%)
Outbuilding roof	21 (6.3%)	44 (12%)	65 (9.3%)
**Inside**			
Interior walls	52 (5.5%)	97 (26.5%)	149 (21.2%)
Ceiling	68 (20.2%)	103 (28.1%)	171 (24.4%)
Interior flooring	65 (19.3%)	122 (33.3%)	187 (26.6%)
Heater flue	24 (7.1%)	53 (14.5%)	77 (11%)
**Overall**			
Any outside ACM ^1^	270 (80.4%)	279 (76.2%)	549 (78.2%)
Any inside ACM	132 (39.3%)	201 (54.9%)	333 (47.4%)
Any ACM	276 (82.1%)	302 (82.5%)	578 (82.3%)

^1^ ACM: asbestos-containing material.

**Table 3:** Priority levels of positive materials inspected in Western Australian houses (n = 336).

Category	Very low	Low	Moderate	High	Total
**Outside**					
Exterior wall cladding	21 (38.9%)	15 (27.8%)	17 (31.5%)	1 (1.9%)	54
Eaves	73 (55.7%)	31 (23.7%)	23 (17.6%)	4 (3.1%)	131
Roof	10 (43.5%)	9 (39.1%)	3 (13%)	1 (4.3%)	23
Gutters	3 (50%)	1 (16.7%)	2 (33.3%)	0	6
Downpipes	22 (71%)	6 (19.4%)	3 (9.7%)	0	31
Backing board to electrical meter box	136 (74.3%)	23 (12.6%)	21 (11.5%)	3 (1.6%)	183
Fencing	76 (44.7%)	44 (25.9%)	42 (24.7%)	8 (4.7%)	170
Outbuilding walls	10 (37%)	8 (29.6%)	9 (33.3%)	0	27
Outbuilding roof	10 (47.6%)	5 (23.8%)	6 (28.6%)	0	21
**Inside**					
Interior walls	25 (48.1%)	14 (26.9%)	13 (25%)	0	52
Ceiling	39 (57.4%)	16 (23.5%)	10 (14.7%)	3 (4.4%)	68
Interior flooring	26 (40%)	12 (18.5%)	16 (24.6%)	11 (16.9%)	65
Heater flue	9 (37.5%)	10 (41.7%)	3 (12.5%)	2 (8.3%)	24
**Overall**					
Outside ACMs ^1^	361 (55.9%)	142 (22%)	126 (19.5%)	17 (2.6%)	646
Inside ACMs	99 (47.4%)	52 (24.9%)	42 (20.1%)	16 (7.7%)	209
**Total**	**460 (53.5%)**	**194 (22.7%)**	**168 (19.6%)**	**33 (3.9%)**	**855**

^1^ ACMs: asbestos-containing material.

**Table 4:** Priority levels of positive materials inspected in other Australian state and territory houses (n = 366).

Category	Very low	Low	Moderate	High	Total
**Outside**					
Exterior wall cladding	32 (44.4%)	15 (20.8%)	20 (27.8%)	5 (6.9%)	72
Eaves	97 (54.2%)	42 (23.5%)	29 (16.2%)	11 (6.1%)	179
Roof	15 (33.3%)	9 (20.0%)	14 (31.1%)	7 (15.6%)	45
Gutters	2 (11.1%)	3 (16.7%)	9 (50.0%)	4 (22.2%)	18
Downpipes	28 (49.1%)	15 (26.3%)	11 (19.3%)	3 (5.3%)	57
Backing board to electrical meter box	105 (62.5%)	34 (20.2%)	25 (14.9%)	4 (2.4%)	168
Fencing	7 (25.9%)	10 (37.0%)	7 (25.9%)	3 (11.1%)	27
Outbuilding walls	19 (34.5%)	15 (27.3%)	16 (29.1%)	5 (9.1%)	55
Outbuilding roof	14 (31.8%)	13 (29.5%)	10 (22.7%)	7 (15.9%)	44
**Inside**					
Interior walls	32 (33.0%)	26 (26.8%)	27 (27.8%)	12 (12.4%)	97
Ceiling	48 (46.6%)	28 (27.2%)	21 (20.4%)	6 (5.8%)	103
Interior flooring	29 (23.8%)	30 (24.6%)	41 (33.6%)	22 (18%)	122
Heater flue	32 (60.4%)	9 (17.0%)	9 (17.0%)	3 (5.7%)	53
**Overall**					
Outside ACMs ^1^	319 (48.0%)	156 (23.5%)	141 (21.2%)	49 (7.4%)	665
Inside ACMs	141 (37.6%)	93 (24.8%)	98 (26.1%)	43 (11.5%)	375
**Total**	**460 (44.2%)**	**249 (23.9%)**	**239 (23.0%)**	**92 (8.8%)**	**1040**

^1^ ACM: asbestos-containing material.

## Supplementary Materials

The following are available online at https://www.mdpi.com/1660-4601/16/24/4922/s1, Table S1: Questions, ratings and descriptive text used in the ACM Check priority assessment, Table S2: Western Australian and other Australian state and territory houses with materials categorised as ’possible’ and ’likely’ for asbestos by ACM Check, Table S3: Summary of priority assessments of positive materials in Western Australian and other Australian houses.
