# Supplementary material for: Using a Mobile Phone App to Identify and Assess Remaining Stocks of In Situ Asbestos in Australian Residential Settings
Source: Int J Environ Res Public Health. 2019 Dec 5;16(24):4922. doi: 10.3390/ijerph16244922 (PMC6950401; doi:10.3390/ijerph16244922)
Supplement: Supplementary file 1 [file ijerph-16-04922-s001.pdf]

Table S1. Questions, ratings and descriptive text used in the ACM Check priority assessment.

Rating	Descriptive text
**Current condition:** *What is the current condition of [insert name of material]?*	
Good	Material is intact (undamaged). There is no or minimal visible water damage, physical damage, or deterioration; no signs of breakdown of any asbestos cement surface through weathering.
Fair	Material has minor damage or deterioration; a few scratches or surface marks; the material is mostly intact; for fencing, slight breakage through plant contact. Any friable (loose or easily crumbled) material is well contained.
Poor	There is moderate breakage, damage or deterioration of materials such as cracks, splits, scratches, panel buckling/distortion, or visible water damage. May have some loose asbestos fibres on the surface of the material. For fencing, there are visible, raised asbestos fibres or moss growth on surfaces due to weathering and age. If you were to touch, gently rub or apply light pressure to the material, the surface material may crumble. Enclosure of any friable material is incomplete or deteriorating.
Very poor	There is major breakage, damage, distortion or deterioration of materials such as multiple major cracks, splits and scratches. Materials such as floor tile extensively damaged and underlying mastic exposed. In the most damaged areas the surface material crumbles very easily upon contact. Any friable material is poorly contained.
**Potential for disturbance:** *What is the likelihood of [insert name of material] being disturbed from access, use, repair, and/or renovation and maintenance activity?*	
Unlikely	Material is unlikely to be disturbed due to no or limited access (i.e. isolated or inaccessible location), no foreseeable need of maintenance or repair, and/or there are no immediate plans for renovation.
Somewhat likely	Material is somewhat likely to be disturbed due to either occasional access (more than once per year but less than monthly), potential need for minor repairs, and/or possible renovations involving the area containing the ACM in the future.
Likely	Material is likely to be disturbed due to either frequent or routine access (i.e. externals of residence and well trafficked areas), likely repairs in the future and/or likely/probable renovations of the area containing the ACM.
Highly likely	Material is highly likely to be disturbed from either frequent or very frequent access (i.e. internals of residence accessed through occupancy, such as kitchens or main bathrooms), planned repairs in the near future, and/or almost certain renovations of the area containing the ACM.
**Priority levels and general recommendations / actions**	
Very low	**Monitor and no immediate action necessary.** Monitoring of potential ACM for any signs of visible deterioration or damage. ACM in good condition may benefit from preventive maintenance actions including painting, sealing or encapsulating.
Low	**Monitor and minor maintenance/repair.** Monitoring of the ACM should be conducted frequently. Maintenance and repair procedures should be considered as a short term measure for any minor damages (sealing cracks and surface scratches, painting the product if appropriate etc.) with plans for removal in the long term (pending on monitoring of the condition of the ACM).
Moderate	**Removal and replacement should be a priority. Major repair activity should be considered as a secondary and temporary action.** Maintenance and repair may be of insufficient benefit. It is recommended that you have the ACM removed and replaced. It is suggested that you consider using the services of a licensed asbestos professional. Depending on the work to be undertaken, this may be either a restricted or unrestricted

	asbestos removal license holder. Further information about asbestos can be found at the WA Health website.
High	**Consult an asbestos professional for removal, disposal and replacement of the ACM.** Removal and replacement of the product as soon as possible is strongly recommended. It is highly recommended that you consult with an asbestos professional, such as an occupational hygienist or a licensed asbestos removalist, for advice on how to deal with the ACM. For certain ACMs (i.e., friable ACMs) that are to be removed, it is strongly recommended that a professional with an Unrestricted Asbestos Removal License obtained from WorkSafe be used to remove and dispose of the material. View a list of Unrestricted Asbestos Removal License holders based in WA.

Table S2. Western Australian and other Australian state and territory houses with materials categorised as 'possible' and 'likely' for asbestos by ACM Check.

	Western Australia (n=336)			Other (n=366)			Total (N=702)		
**Category**	**Negative**	**Possible**	**Likely**	**Negative**	**Possible**	**Likely**	**Negative**	**Possible**	**Likely**
**Outside**									
Exterior wall cladding	282 (83.9%)	0	54 (16.1%)	294 (80.3%)	0	72 (19.7%)	576 (82.1%)	0	126 (17.9%)
Eaves	205 (61.0%)	30 (8.9%)	101 (30.1%)	187 (51.1%)	36 (9.8%)	143 (39.1%)	392 (55.8%)	66 (9.4%)	244 (34.8%)
Roof	313 (93.2%)	0	23 (6.8%)	321 (87.7%)	0	45 (12.3%)	634 (90.3%)	0	68 (9.7%)
Gutters	330 (98.2%)	0	6 (1.8%)	348 (95.1%)	0	18 (4.9%)	678 (96.6%)	0	24 (3.4%)
Downpipes	305 (90.8%)	9 (2.7%)	22 (6.5%)	309 (84.4%)	24 (6.6%)	33 (9.0%)	614 (87.5%)	33 (4.7%)	55 (7.8%)
Backing board to electrical meter box	153 (45.5%)	33 (9.8%)	150 (44.6%)	198 (54.1%)	41 (11.2%)	127 (34.7%)	351 (50.0%)	74 (10.5%)	277 (39.5%)
Fencing	166 (49.4%)	31 (9.2%)	139 (41.4%)	339 (92.6%)	13 (3.6%)	14 (3.8%)	505 (71.9%)	44 (6.3%)	153 (21.8%)
Outbuilding walls	309 (92.0%)	14 (4.2%)	13 (3.9%)	311 (85.0%)	12 (3.3%)	43 (11.7%)	620 (88.3%)	26 (3.7%)	56 (8.0%)
Outbuilding roof	315 (93.8%)	0	21 (6.3%)	322 (88.0%)	0	44 (12.0%)	637 (90.7%)	0	65 (9.3%)
**Inside**									
Interior walls	284 (84.5%)	0	52 (15.5%)	269 (73.5%)	0	97 (26.5%	553 (78.8%)	0	149 (21.2%)
Ceiling	268 (79.8%)	0	68 (20.2%)	263 (71.9%)	0	103 (28.1%)	531 (75.6%)	0	171 (24.4%)
Interior flooring	271 (80.7%)	23 (6.8%)	42 (12.5%)	244 (66.7%)	24 (6.6%)	98 (26.8%)	515 (73.4%)	47 (6.7%)	140 (19.9%)
Heater flue	312 (92.9%)	19 (5.2%)	5 (1.5%)	313 (85.5%)	27 (7.4%)	26 (7.1%)	625 (89.0%)	46 (6.6%)	31 (4.4%)
**Total (all materials)[1]**	**3513 (80.4%)**	**159 (3.6%)**	**696 (15.9%)**	**3718 (78.1%)**	**177 (3.7%)**	**863 (18.1%)**	**7231 (79.2%)**	**336 (3.7%)**	**1559 (17.1%)**

[1]Excluding wall tile backing

Table S3. Summary of priority assessments of positive materials in Western Australian and other Australian houses.

Factor	Western Australia (n=855)		Other (n=1040)		Total (N=1895)	
	n	%	n	%	n	%
**Current condition**						
Good	353	41.3%	391	37.6%	744	39.3%
Fair	357	41.8%	403	38.8%	760	40.1%
Poor	121	14.2%	167	16.1%	288	15.2%
Very poor	24	2.8%	79	7.6%	103	5.4%
**Potential for disturbance**						
Unlikely	416	48.7%	428	41.2%	844	44.5%
Somewhat likely	283	33.1%	317	30.5%	600	31.7%
Likely	81	9.5%	154	14.8%	235	12.4%
Highly likely	75	8.8%	141	13.6%	216	11.4%
**Priority level**						
Very low	460	53.8%	460	44.2%	920	48.5%
Low	194	22.7%	249	23.9%	443	23.4%
Moderate	168	19.6%	239	23.0%	407	21.5%
High	33	3.9%	92	8.8%	125	6.6%
**Total positive materials**	**855**	**100.0%**	**1040**	**100.0%**	**1895**	**100.0%**

[1]Excluding wall tile backing
